# Biotransformation of obefazimod, a novel potential anthelmintic, in sheep and the target nematode *Haemonchus contortus*

**DOI:** 10.1038/s41598-026-49484-1

**Published:** 2026-04-22

**Authors:** Lukáš Lochman, Martin Novák, Lenka Skálová, Gabriela Svobodová, Radim Kučera, Lucie Raisová Stuchlíková

**Affiliations:** 1 Department of Pharmaceutical Chemistry and Pharmaceutical Analysis, Faculty of Pharmacy in Hradec Králové, Charles University, Akademika Heyrovského 1203, 500 05 Hradec Králové, Czech Republic; 2 Biomedical Research Centre, University Hospital Hradec Králové, Sokolská 581, 500 05 Hradec Králové, Czech Republic; 3 Department of Biochemical Sciences, Faculty of Pharmacy in Hradec Králové, Charles University, Akademika Heyrovského 1203, 500 05 Hradec Králové, Czech Republic

**Keywords:** Obefazimod (ABX464), Anthelmintics, Biotransformation, *Haemonchus contortus*, Chromatography-mass spectrometry, Biochemistry, Computational biology and bioinformatics, Diseases, Drug discovery, Microbiology

## Abstract

Parasitic nematodes cause significant health issues in livestock, resulting in clinical complications and economic losses worldwide. As the efficacy of current anthelmintic drugs is being compromised by the emergence of drug resistance, a search for novel therapeutics with new modes of action is necessary. Obefazimod (ABX464) originally developed for human inflammatory diseases, has recently shown promising nematocidal activity against *Haemonchus contortus* and other parasitic nematodes. This study aimed to elucidate the biotransformation pathways of obefazimod in *H. contortus* and in its host, sheep. Adult nematodes (females and males separately) of drug-susceptible and drug-resistant strains and ovine liver models were used. Using a newly developed and optimized UHPLC-HRMS/MS method, we identified 13 metabolites formed via phase I and II metabolism. These findings provide the first insight into the metabolic fate of obefazimod in target parasites and host species.

**Supplementary Information:**

The online version contains supplementary material available at https://doi.org/10.1038/s41598-026-49484-1.

## Introduction

Diseases caused by parasitic nematodes in livestock are associated with various clinical complications in animals and economic losses to farmers^1^. Although pharmacotherapy as a basic strategy for the treatment of nematodes in livestock is vital for efficient and welfare-friendly production, the effectiveness of available anthelmintics has become limited due to increasing drug resistance in nematode populations^2,3^. Therefore, considerable efforts have been devoted to the development of new anthelmintic drugs, especially those with new molecular targets and more pronounced efficacy against nematodes resistant to classical anthelmintics^4^.

Several approaches are used to identify new anthelmintics^5^. Among these, drug repurposing, also called “therapeutic switching”, is an up-to-date strategy based on the novel indication of an approved drug. The advantage of this strategy is the availability of preclinical and clinical data, which might accelerate drug development owing to lower costs and reduced risk of toxicity-related failure^6^.

Recently, a screening of the compounds used in human clinical trials within the ‘Pandemic Response Box’ (from Medicines for Malaria Venture) against the model nematode *Caenorhabditis elegans* uncovered the anthelmintic activity of a quinoline derivative, obefazimod (OFM; ABX464). OFM, originally conceived as a potential human immunodeficiency virus infection drug, is now in phase III clinical trials as a potential drug for the treatment of ulcerative colitis^7^. In phase II clinical trials, OFM significantly proved efficacy in patients with ulcerative colitis and an overall good safety profile^8,9^. Moreover, OFM exhibited significant efficacy in patients with active rheumatoid arthritis^10^. In addition, OFM is now a nematocidal candidate with a significant activity against *Caenorhabditis elegans* and *Haemonchus contortus* (barber’s pole worm), a highly pathogenic parasite of ruminant livestock, and moderate activity against other parasitic nematodes (including *Ancylostoma*, *Heligmosomoides,* and *Strongyloides* species)^11–13^. Concerning the nematocidal activity of OFM, protein HCON_00074590 (a predicted aldo–keto reductase) was identified as the OFM molecular target in *H. contortus*^11^, while eight different proteins interacted with OFM in *C. elegans*^12^. On the other hand, OFM activity in humans is attributed to specific upregulation of miR-124 (a crucial modulator of inflammation and innate immunity)^14^.

A pharmacokinetic study in human volunteers showed that OFM was rapidly and substantially metabolized to the main metabolite OFM-*N*-glucuronide^15^. However, except for this human metabolite, no further information about OFM biotransformation is available yet. From the perspective of the potential use of OFM as an anthelmintic, the detailed study of its biotransformation in parasitic nematodes, as well as in their host, using Ultra-High Performance Liquid Chromatography-High Resolution Mass Spectrometry (UHPLC-HRMS) is essential. UHPLC-HRMS combines the advantages of rapid and effective separation of the parent compound from its metabolites with accurate mass-to-charge ratio measurements and fragmentation studies to predict the elemental composition of metabolites^16,17^. All these benefits should lead to the meaningful design of metabolite structures.

The present study was designed to elucidate OFM biotransformation in *H. contortus,* as this is necessary to determine the ability of this parasite to protect itself against OFM. OFM biotransformation was compared in *H. contortus* adults of an ISE (Inbred-Susceptible-Edinburg, MHco3) strain, which is susceptible to all main classes of anthelmintics^18^, and a multi-resistant WR (White River; MHco4) strain, which is resistant to multiple classes of anthelmintics, particularly benzimidazoles, imidazothiazoles, and macrocyclic lactones^19^. With respect to potential sex-differences in OFM biotransformation, females and males were investigated separately. As the sheep is intended target species, OFM biotransformation has also been investigated in an in vitro models of ovine liver. A new UHPLC-HRMS method was developed to assess OFM biotransformation in the parasitic nematode and its host, to suggest the structures of the resulting metabolites, and to determine their relative abundances. Nevertheless, anthelmintic activity and biological relevance of these metabolites require further investigation.

## Materials and methods

### Chemicals and reagents

The OFM was a kind gift from Prof. R.B. Gasser (The University of Melbourne, Australia). Liquid sterile-filtered medium RPMI-1640 medium, Williams’ E medium, and other chemicals of LC–MS grade were obtained from Sigma-Aldrich (St. Louis, MO, USA). Monepantel was purchased from LGC standards S.r.l. (Sesto San Giovanni, Italy). Chromatographic columns: Hypersil GOLD C18 column 100 × 4.6 mm, particle size 3.0 µm (Thermo Fisher Scientific, Brno, Czech Republic), Arion Polar C18 column 150 × 3.0 mm, particle size 3.0 µm (Chromservis, Prague, Czech Republic), and ACE Excel C18-PFP column 100 × 3.0 mm, particle size 3.0 µm and 100 × 2.1 mm, particle size 1.7 µm (VWR, Stříbrná Skalice, Czech Republic).

### Experimental animals

The lambs (6–7 months of age, originated from Dibaq a.s.) were bred and slaughtered in agreement with Czech slaughtering regulations for farm animals and the Protection of Animals from Cruelty Act No. 246/1992, Czech Republic. The breeding facility was accredited by the Ministry of Agriculture of the Czech Republic for experimental sheep housing (Approval MZE-53255/2022-13143). The experimental project was evaluated and approved by the Ethics Committee of the Ministry of Education, Youth and Sports (Project number MSMT-20144/2023-4). The abomasum was kept in warm water (37 °C) and transported to the laboratory. One lobe of the liver was washed with a chilled Euro Collins solution (15 mM KH_2_PO_4_, 42.5 mM K_2_HPO_4_, 15 mM KCl, 10 mM NaHCO_3_, and 0.2 M glucose) and transported to the laboratory in a chilled container.

### Preparation of the primary culture of hepatocytes

After removal of the liver from the abdominal cavity of the lamb, the liver was flushed through the main veins with Euro Collins solution (15 mM KH_2_PO_4_, 42.5 mM K_2_HPO_4_, 15 mM KCl, 10 mM NaHCO_3_, and 0.2 M glucose). The hepatocytes were obtained from the liver by the two-step collagenase method^20^. Briefly, the part of the left lobe was perfused with a salt solution (0.14 M NaCl, 5.0 mM KCl, 0.8 mM MgSO_4_) in a Na^+^/K^+^ phosphate buffer (0.2 mM, pH 7.40) containing a calcium-binding component (0.4 mM EGTA). Consequently, the liver tissue was perfused with a phosphate buffer solution containing calcium chloride (1.46 mM) and collagenase (30 mg/100 mL) at 37 °C. The collagenase perfusion proceeded for 5–10 min, depending on the digestion rate. Following perfusion, the hepatocytes were released into a phosphate buffer solution containing calcium chloride and bovine serum albumin (BSA). The obtained suspension was filtered through a nylon mesh and centrifuged (40 g, 5 min, 4 °C). The pellet was resuspended in a chilled buffer solution, after which the washing procedure was repeated twice. Finally, suspensions containing one million viable (75–80%) cells in 1 mL of ISOM culture medium (composed of 1:1 *(v/v)* mixture of Ham’s F12 and Williams’ E medium) containing 5% fetal calf serum were placed into 60 mm plastic dishes pre-coated with collagen or into 96-well plates. The cultures were maintained without the substrate for 4 h at 37 °C in a humid atmosphere of air and 5% CO_2_.

### Preparation of precision-cut liver slices (PCLS)

Cores with a diameter of 8 mm were punched out of the liver tissue. PCLS were prepared in ice-cold Krebs–Henseleit buffer supplemented with 25 mM glucose, 25 mM NaHCO_3_, 10 mM HEPES, and saturated with carbogen using a Krumdieck tissue slicer MD4000 (Alabama Research and Development, Munford, AL, USA). PCLS (diameter: 8 mm, thickness: 150–170 μm⁏ wet weight 14.4 ± 2.1 mg) were placed individually in 12-well plates containing Williams’ Medium E with L-glutamine, supplemented with 25 mM glucose (final concentration 36 mM) and 50 μg mL^−1^ gentamycin at 37 °C with continuous shaking (90 rpm).

### Collection of nematodes

*H. contortus* from ISE strain (Inbred-Susceptible-Edinburg, MHco3), which is susceptible to all main classes of anthelmintics^18^, and a multi-resistant WR (White River; MHco4) strain^19^, both obtained from the Moredun Research Institute (Scotland, UK), were used in this study. Six parasite-free lambs (3–4 months old) were orally infected with 5,000 third-stage larvae (L3) of *H.* *contortus*. Six weeks after infection, the animals were stunned, exsanguinated, and the abomasum was transported to the laboratory in warm water (37 °C). The adult worms were harvested using the agar method^21^ and washed in phosphate buffer saline. The *H. contortus* males and females were manually separated under a microscope and immediately used for incubation or for homogenate preparation.

### Incubation of biological materials with OFM

In the case of hepatocytes, ISOM medium with fetal bovine serum (FBS) was removed, and hepatocytes attached to 60-mm dishes pre-coated with collagen were covered with fresh serum-free ISOM medium (3 mL) with OFM (10 µM). The OFM was pre-dissolved in DMSO. The final concentration of DMSO in the medium did not exceed 0.1%. After 24 h, the hepatocytes were scraped and stored at − 80 °C.

The PCLS, after 1 h of pre-incubation in 1 mL of Williams’ Medium E with L-glutamine, supplemented with 25 mM glucose (final concentration 36 mM) and 50 μg mL^−1^ gentamycin, were transferred to new 12-well plates containing 1.3 mL of fresh medium (the same type) with 10 µM OFM (pre-dissolved in DMSO) and subsequently incubated for 24 h at 37 °C under a continuous supply of 80% O_2_ and 5% CO_2_ with shaking (90 rpm). The final concentration of DMSO in the medium did not exceed 0.1%. After the incubation, the medium was transferred to plastic tubes. The PCLS were washed in phosphate-buffered saline (PBS, pH 7.40) and transferred into plastic tubes. The samples were frozen and stored at − 80 °C.

The freshly isolated living *H.* *contortus* adults were washed three times in PBS (pH 7.40). Males and females (15 and 10 per one well, respectively) were placed in 12-well plates containing 1 mL of RPMI medium (pH 7.40, containing 60 µg mL^−1^ penicillin, and 100 µg mL^−1^ streptomycin) and cultivated at 37 °C in a humid atmosphere with 5% CO_2_. At the beginning of the incubation, 0.5 mL of the medium from each well containing nematodes was removed and replaced with an equal volume of fresh medium containing 10 µM OFM (pre-dissolved in DMSO). The final concentration of DMSO in the medium was 0.1% (*v/v*). After a 24-h incubation, the medium was transferred to plastic tubes. The nematodes were washed three times in PBS (pH 7.40) and were also transferred to plastic tubes. The samples were frozen and stored at − 80 °C.

PCLS samples were performed in six biological replicates from ovine 1 and 2, and subsequently presented as an average value. All other samples were performed in three biological replicates. In all incubations, chemical blank samples (medium with OFM, without biological materials) and the biological blank samples (medium with biological materials, without OFM) were prepared in the same way. The protein concentration of the samples was measured using a bicinchonic acid assay according to the manufacturer’s protocol provided by Sigma-Aldrich (Prague, Czech Republic).

### Sample homogenization and extraction

Hepatocytes (frozen after incubation) were thawed and suspended in 1 mL of 0.1 M PBS (pH 7.40). The suspension was homogenized using a Sonopuls sonicator (Bandelin, Germany) with four cycles of 30 s sonication, each followed by 60 s cooling on ice. The homogenate was centrifuged (3,000 g, 5 min), and the supernatant was collected for further processing.

PCLS samples (frozen after incubation) were thawed and mixed with 750 µL of 0.1 M PBS (pH 7.40). Then, the homogenization using glass beads and a FastPrep-24™ 5G Instrument (MP Biomedicals, Santa Ana, CA, USA) was performed. The homogenate was centrifuged (3,000 g, 5 min) to obtain the supernatant.

Nematodes (frozen after incubation) were thawed and suspended in 750 µL of chilled 0.1 M PBS (pH 7.40) and homogenized (eight cycles, each consisting of 2 × 20 s sonication followed by 40 s cooling on ice) using a Sonopuls sonicator (Bandelin, Germany). The homogenate was centrifuged (3,000 g, 5 min), and the supernatant was collected.

For all sample types, 50 µL of the obtained solution was mixed with ACN (150 µL) containing 1 µM monepantel (internal standard; IS). After that, the mixture was shaken (800 rpm, 10 min) and centrifuged (30,000 g, 15 min, 20 °C). The supernatant was collected and stored at − 20 °C. Before UHPLC-MS/MS analysis, all samples were thawed and filtered using Millex PVDF filters (0.22 µm, Merck Millipore, Germany).

#### Analytical conditions of UHPLC-MS/MS

The samples were measured by UHPLC-MS (full-scan) and UHPLC-MS/MS (parallel reaction monitoring, PRM) methods using a Dionex UltiMate 3000 RS LC system coupled to a hybrid Q Exactive Plus mass spectrometer (all Thermo Fisher Scientific, Bremen, Germany). Samples were injected into ACE Excel C18-PFP column (100 × 2.1 mm, particle size 1.7 µm). A gradient elution was employed, with mobile phase A consisting of an aqueous solution of 0.1% formic acid (v/v), and mobile phase B comprising acetonitrile with 0.1% formic acid (v/v). The method optimization resulted in the following gradient profile: 0–0.3 min 5% B; 0.3–11 min 5% B → 100% B; 11–12.5 min 100% B. After that, the column was equilibrated to the initial conditions for 3 min. The flow rate of 0.35 mL/min and temperature of 30 °C were set. The sample injection volume was 1 µL. Positive and negative ionization modes were used to aquire full-scan data. All obtained MS spectra were correlated with blank samples and analyzed using XCalibur software (ver. 4.3.73.11.)^22^. Identification was achieved in PRM mode using the following parameters: NCE = 10–70 (chosen individually according to the fragmentation stability of metabolites, see Figs. S1–S3 in Supplementary materials)⁏ spray voltage = 3.5 kV; capillary temperature = 300 °C; sheet gas = 55 arbitrary units; auxiliary gas = 15 arbitrary units; spare gas = 3 arbitrary units; probe heater temperature = 250 °C; maximum spray current = 100 µA; S-lens RF level = 50, inclusion list: *m/z* values of parent compounds and metabolites of interest in the range ± 0.01 *m/z*.

## Results and discussion

Comprehensive information on drug biotransformation is important to ensure drug efficacy and safety, as well as to limit potential drug-drug interactions. Two in vitro ovine liver models (primary culture of hepatocytes and PCLS) and adult nematodes (females and males separately) of ISE and WR strains were used for this purpose.

A new sensitive UHPLC-Q-Exactive-MS/MS method for the identification and subsequent quantification of OFM metabolites was developed and optimized for monitoring OFM biotransformation. Three C18 stationary phases with modified functionalities (Hypersil GOLD C18, ACE Excel C18-PFP, and Arion Polar C18) were tested and evaluated based on their chromatographic performance, including resolution, peak symmetry, and retention properties. The ACE Excel C18-PFP phase, selected due to its favorable peak shape characteristics, provided superior separation efficiency and selectivity for all detected metabolites. This phenomenon was probably due to the advantageous connection of the C18 and PFP stationary phase combined in one column. The mobile phase consisted of 0.1% (v/v) formic acid in water (mobile phase A) and 0.1% (v/v) formic acid in acetonitrile (mobile phase B) was applied in a gradient mode (see chapter: Analytical conditions of UHPLC-MS/MS). Optimal separation was achieved at a column temperature of 30 °C. The final method employed an ACE Excel C18-PFP column with reduced dimensions (100 × 2.1 mm, particle size 1.7 µm) and a flow rate of 0.35 mL/min, enabling baseline separation of all analytes within 15 min. Positive ionization provided a better signal-to-noise ratio for OFM and its metabolites than negative ionization. Therefore, all the results presented in this study were calculated using data acquired in positive ESI mode. The structures of metabolites were suggested based on their accurate *m/z* values, isotopic distribution, and fragmentation spectra. High-resolution mass spectrometry measurements allow the design of metabolites’ elemental composition, and the type of metabolic reactions was suggested according to exact mass defects.

A limitation of this study represents a lack of metabolite standards needed for quantification. Therefore, metabolites were quantified relatively using the ratio between the metabolite peak area and the IS peak area. Monepantel was chosen as a suitable IS, as its physicochemical properties are reasonably comparable to those of OFM. The ratios of peak areas were subsequently normalized to the protein content of each sample.

**Fragmentation of OFM.**

**Fig. 1:**
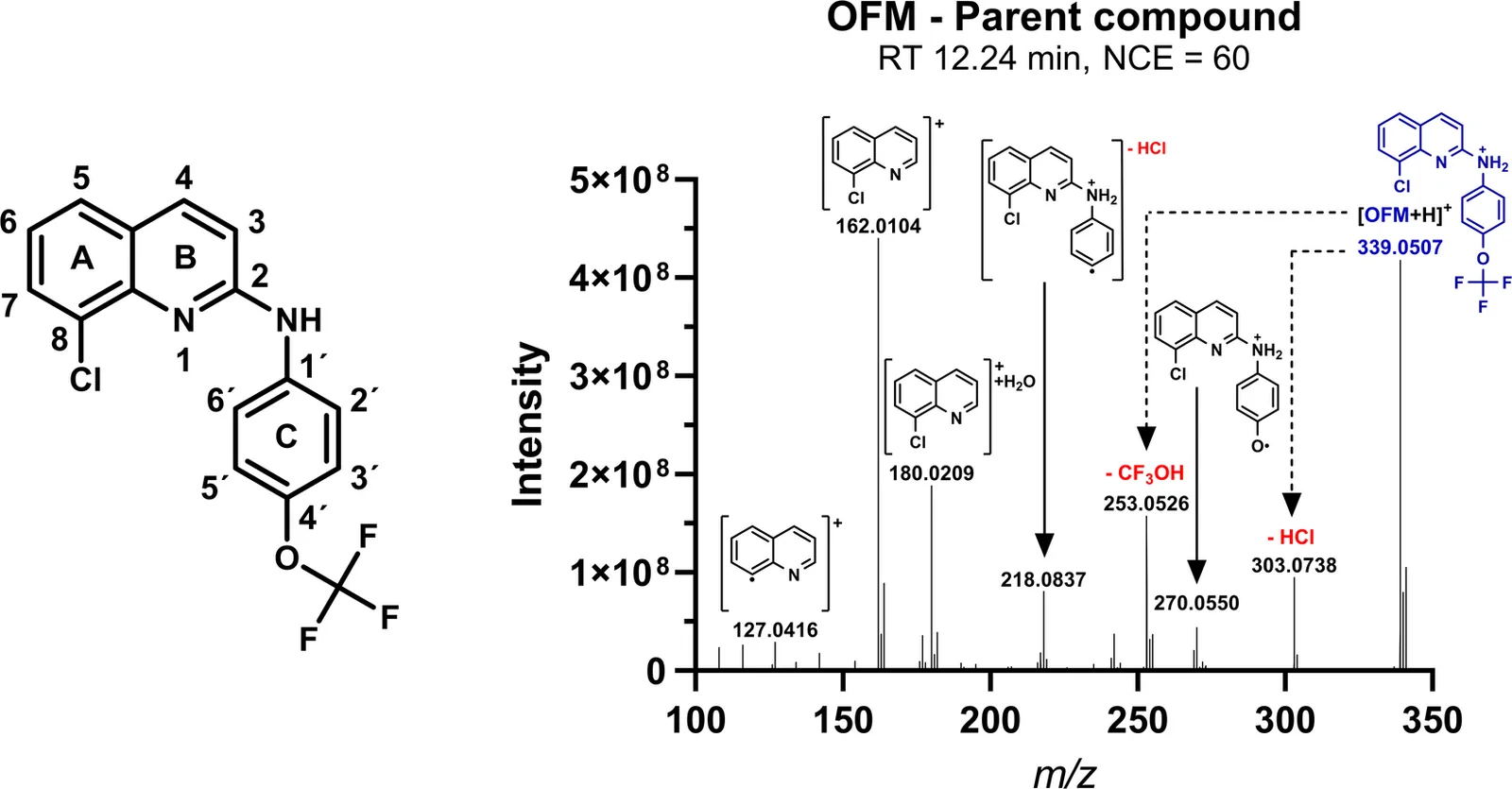
The chemical structure of OFM with proposed aromatic rings labeling, atom numbering scheme, and experimentally obtained fragmentation spectrum of the parent compound with the interpretation of fragments.

 The obtained MS/MS spectra and the proposed fragmentation of OFM in positive ion mode are shown in Fig. 1. The OFM was detected at *m/z* 339.0507 [M + H]^+^ (t_R_ = 12.24 min). Its fragmentation yielded a characteristic product ion at *m/z* 303.0738 (neutral loss (NL) of HCl), which is typical for halogenated derivatives^23^. The product ions at *m/z* 270.0550 (radical loss of the CF_3_ group) and *m/z* 253.0526 (NL of CF_3_OH) are attributed to cleavage of the trifluoromethoxy group, while the ion at *m/z* 218.0837 corresponds to a fully dehalogenated radical cation. Among other fragments belong *m/z* 180.0209, *m/z* 162.0104, and *m/z* 127.0416 that correspond to fragments of the quinoline ring.

**The biotransformation of OFM in H. contortus and ovine liver.**

**Fig. 2:**
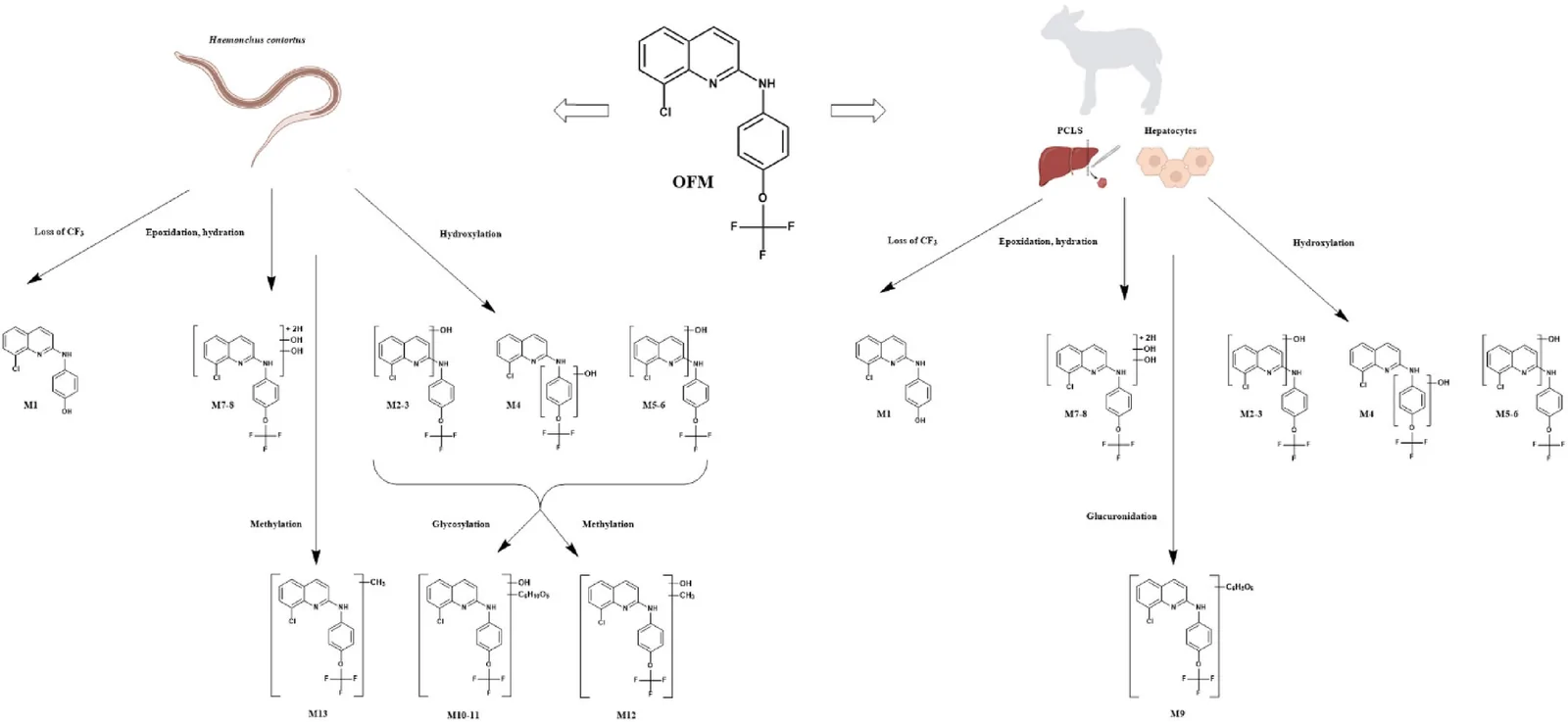
Proposed metabolic pathway of OFM in parasitic nematode *H. contortus* and in vitro ovine liver models.

 Twelve metabolites of OFM formed in *H. contortus,* and eleven metabolites in ovine liver were found. Identification of their structures revealed that the enzymatic systems of the tested species biotransform OFM via several reactions of phase I and II. The description of all detected metabolites (retention times, experimental and theoretical *m/z* values, and elemental compositions) together with their product ions is presented in Tables 1 and S1. The proposed OFM metabolic pathways are illustrated in Fig. 2. All MS/MS spectra of the detected metabolites are provided in the Supplementary materials (Figs. S1–S3).

**Table 1:** Analytical description of OFM and its identified metabolites containing details regarding their designation, retention times, experimental *m/z* values of [M + H]^+^ in ESI positive-ion mode, mass accuracies, elemental composition, and description of metabolic reaction. * M12 and M13 were proposed only based on the exact mass. Confirmation by fragmentation spectra was not possible due to the low concentrations.

Designation	t_R_ (min)	Detected *m/z* values [M + H]^+^	Exact mass shift (ppm)	Elemental composition	Metabolic reaction	
					Phase I	Phase II
OFM	12.24	339.0507	0.00	C_16_H_11_ClF_3_N_2_O	–	–
M1	7.08	271.0632	− 0.37	C_15_H_12_ClN_2_O	Loss of CF_3_	–
M2	10.68	355.0455	− 0.28	C_16_H_11_ClF_3_N_2_O_2_	Hydroxylation	–
M3	10.90	355.0455	− 0.28	C_16_H_11_ClF_3_N_2_O_2_	Hydroxylation	–
M4	11.27	355.0455	− 0.28	C_16_H_11_ClF_3_N_2_O_2_	Hydroxylation	–
M5	11.62	355.0455	− 0.28	C_16_H_11_ClF_3_N_2_O_2_	Hydroxylation	–
M6	11.73	355.0454	− 0.56	C_16_H_11_ClF_3_N_2_O_2_	Hydroxylation	–
M7	9.00	373.0568	1.88	C_16_H_13_ClF_3_N_2_O_3_	Epoxidation, hydration	–
M8	9.09	373.0561	0.00	C_16_H_13_ClF_3_N_2_O_3_	Epoxidation, hydration	–
M9	8.08	515.0826	− 0.19	C_22_H_19_ClF_3_N_2_O_7_	–	Glucuronidation
M10	8.45	517.0984	0.00	C_22_H_21_ClF_3_N_2_O_7_	Hydroxylation	Glycosylation
M11	9.15	517.0985	0.19	C_22_H_21_ClF_3_N_2_O_7_	Hydroxylation	Glycosylation
M12*	12.37	369.0611	− 0.27	C_17_H_13_ClF_3_N_2_O_2_	Hydroxylation	Methylation
M13*	12.54	353.0663	0.00	C_17_H_13_ClF_3_N_2_O	–	Methylation

**Phase I biotransformation of OFM.**

 The phase I metabolic pathway of OFM includes hydroxylation, epoxidation accompanied by hydration, and the loss of the trifluoromethyl (CF_3_) group. These transformations are generally mediated by cytochrome P450 enzymes (CYPs), which introduce polar functional groups and thereby increase the solubility of the parent compound and facilitate its subsequent conjugation in phase II metabolism^24^. CYPs are known to be highly conserved across taxa^25^. In nematodes such as *Caenorhabditis elegans*, extensive CYP gene families have been identified, some of which are functionally analogous to mammalian enzymes^26^. In addition to CYPs, dehalogenation, a key reaction in the detoxification of halogenated compounds, can be catalyzed by NADPH–cytochrome P450 reductase. This reaction involves the reduction of carbon–halogen bonds, leading to the formation of less persistent and more readily metabolized intermediates^27^.

The first identified metabolite **M1** with *m/z* 271.0632 [M + H]^+^ was detected at 7.08 min and contaning OH instead of the O-CF_3_ group (Δ *m/z* − 67.9874). The fragmentation spectrum (Fig. S1) shows high similarity with the parent compound (Fig. 1). The fragment at *m/z* 253.0524 is identical to the OFM fragment and corresponds to the NL of water. Another fragment, which corresponds to the NL of HCl, was found at *m/z* 235.0863, whereas *m/z* 218.0839 represents a completely dehalogenated radical cation. The ions at *m/z* 180.0209, *m/z* 162.0103, and *m/z* 127.0415 correspond to fragmentation ions previously detected in the parent compound.

Metabolites (**M2–M6**) were formed via a hydroxylation step at five different substitution positions at *m/z* 355.046 [M + H]^+^ (t_R_ = 10.68–11.73 min). The characteristic fragmentation ions were produced via NL of the HCl (*m/z* at 319.069), and NL of CF_3_OH (*m/z* at 269.047), radical loss of the CF_3_ group (*m/z* at 286.050), and complete dehalogenation (*m/z* at 234.078/233.071) (Fig. S1). Generally, the position of hydroxylation is influenced by various factors related to the substrate’s chemical and electrone properties, and the enzyme system. CYPs superfamily are the main enzymes that could catalyze OFM hydroxylation. The specificity of substrate hydroxylation by CYPs is determined specifically by three factors: the affinity of the substrate for the CYP active site, the intrinsic reactivity of the individual C–H bonds, and the spatial orientation of the substrate within the enzyme’s active site^28^.

Due to the non-specific fragmentation spectra, the availability of chemical standards of metabolites is necessary to specify the exact position of hydroxylation. However, some differences were observed between experimental fragmentation spectra of **M4** and other hydroxylated metabolites. Firstly, the precursor ion was less stable in the case of **M4**. Secondly, different product ions were found. The product ions at *m/z* 196.016, *m/z* 178.005, and *m/z* 142.029/143.037 were present only in **M2, M3, M5**, and ion *m/z* 194.0240 in **M6**, whereas these were not produced by fragmentation of **M4**. Considering the OFM structure, we assume that hydroxylation occurred on the quinoline ring in the case of **M2, M3, M5, M6,** and on the C-ring in the case of **M4,** as the typical product ions *m/z* 178.0291 and *m/z* 162.0104 were observed in the fragmentation spectrum of **M4**. However, for definitive confirmation of the hydroxylation positions, it would be necessary to have the appropriate chemical standards available^28^.

Two metabolites, **M7** and **M8** (t_R_ = 9.00 and 9.09 min, *m/z* 373.0568 and *m/z* 373.0561 [M + H]^+^) with Δ *m/z* 34.0054 indicate the epoxidation followed by hydration at two different sites of OFM (Fig. S2). Fragmentation yielded the most intense product ions: at *m/z* 355.0456, which corresponds to NL of water (Δ *m/z* 18.0106); at *m/z* 337.0793, representing NL of HCl, and at *m/z* 319.0689, combining NL of water and HCl. Additionally, these metabolites provided another product ions at *m/z* 327.051, *m/z* 309.0844, and *m/z* 291.0738/292.0819 which represent cleavage of the aromatic ring, and *m/z* 320.0761, which corresponds to the radical loss of the chlorine atom. The fragments with *m/z* lower than 250 indicate other non-specific cleavage processes (Fig. S2). Similarly to the hydroxylated metabolites, it was not possible to determine the exact biotransformation position due to the non-specific fragmentation spectra. This process, typical for aromatic or heterocyclic compounds, is in mammalian liver catalyzed by CYPs and epoxide hydrolases^24^.

In both tested species, all three types of phase I reactions were consistently detected, indicating that both organisms possess a complete enzymatic apparatus capable of converting xenobiotics through standard biochemical pathways. Hydroxylation was identified as the main biotransformation step, followed by epoxidation coupled with hydration and supplemented by the cleavage of the CF_3_ group in both species. Presence of comparable metabolic routes in parasitic nematodes suggests that these organisms have evolved enzyme systems capable of biotransformation of a wide range of xenobiotics^29,30^. If nematodes have similar biotransformation pathways to ruminants, then metabolites formed in the parasite might resemble those formed in the host, making it more difficult to distinguish between host- and parasite-derived metabolic profiles^31^.

**Fig. 3:**
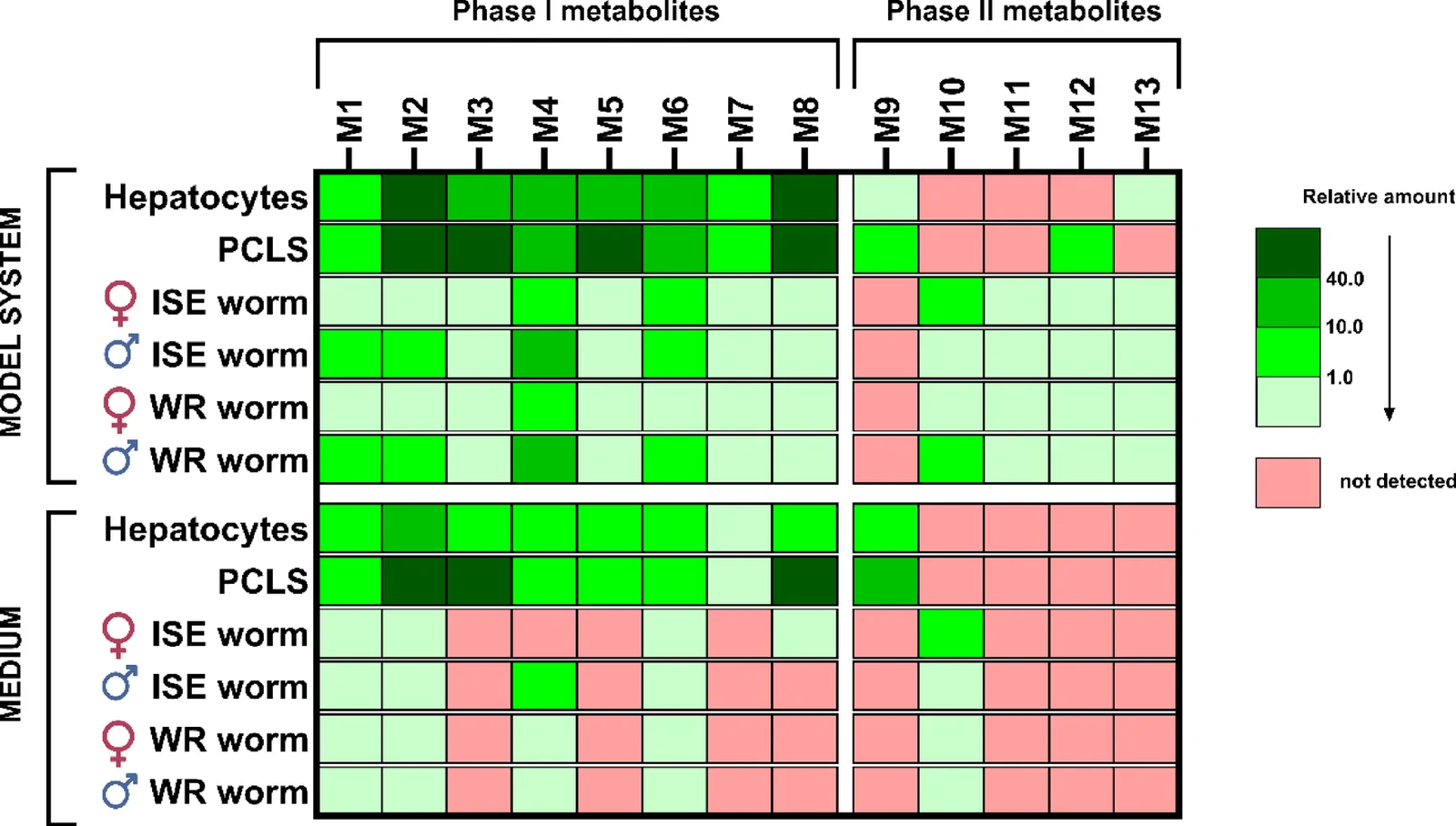
The concentrations of suggested metabolites using in vitro ovine liver models, i.e., ovine hepatocytes and precision-cut liver slices (PCLS), and *H. contortus* nematodes, i.e., Inbred-Susceptible-Edinburg, MHco3 strain (ISE) and multi-resistant White River, MHco4 strain (WR). The presence of metabolites is indicated by green fields with increasing color intensity corresponding to rising concentration. The amount of metabolites is relatively expressed as a ratio between each metabolite’s peak area and the peak area of the internal standard (IS) monepantel. Presented values were normalized to 1 mg of protein content.

Comparing epoxidation accompanied by hydration in sheep and nematode (Figs. 3, S4), the ratio **M7/M8** was different in nematodes and ovine liver models, where M8 was predominantly formed. Concerning hydroxylation, the nematodes produced 5 different hydroxy metabolites, similar to the ovine liver models. Nematodes yielded predominantly metabolites **M4** and **M6**. However, the production of **M3** and **M5** was considerably reduced in contrast to the ovine liver models. The observed differences in metabolite formation among nematodes and ovine liver models probably result from variations in the enzymatic activity and substrate specificity of their phase I biotransformation enzymes. Comparing *H. contortus* males and females, the males were found to be more active in OFM phase I biotransformation (Figs. 3, S4). Such findings align with studies in mammals and invertebrates showing that sex-specific expression of metabolic enzymes can affect xenobiotic processing^32^. On the other hand, no significant differences were observed in OFM phase I biotransformation in drug-susceptible and drug-resistant strains of *H. contortus*. The differences in the presence and amounts of identified metabolites among all tested samples are depicted in Fig. 3; for details see Supplementary materials (Fig. S4).

**Phase II biotransformation of OFM.**

 The glucuronidation, glycosylation, and methylation represented products of OFM phase II biotransformation. Glucuronidation, catalyzed by UDP-glucuronosyltransferases, is one of the most prominent detoxification pathways in mammals and serves as the main mechanism for deactivating a wide range of drugs and environmental contaminants. Glycosylation, commonly mediated by UDP-glucose-dependent glycosyltransferases, increases the polarity of xenobiotics, promoting their elimination via bile or urine. Methylation, catalyzed by methyltransferases using S-adenosylmethionine as a methyl donor, reduces the reactivity of compounds and often prevents their interaction with cellular targets^24^.

The metabolite **M9** was eluted at 8.08 min and detected at *m/z* 515.0826 [M + H]^+^ as OFM *N*-glucuronide. The fragmentation spectra (Fig. S3) displayed the characteristic NL of glucuronide Δ*m/z* 176.0320 (*m/z* 339.0534)^33,34^.

Two glycosides of OFM (**M10, M11**) were detected at *m/z* 517.0984 and *m/z* 517.0985 [M + H]^+^, and were eluted at 8.45 and 9.15 min, respectively. The characteristic NL for a hexose Δ *m/z* 162.0528 (*m/z* 355.045) was observed (Fig. S3).

Also, two minor methylated OFM metabolites were found: **M13** (12.54 min) at *m/z* 353.0663 [M + H]^+^ and **M12** (12.37 min, hydroxylation followed by methylation) at *m/z* 369.0611 [M + H]^+^. This reaction is most likely catalyzed by the enzyme nicotinamide *N*-methyltransferase, which has been identified in ruminant species^35^. The position of methylation depends on the fragmentation pattern. However, no fragmentation spectra were obtained for these metabolites because of their low abundance. Both methylated metabolites of OFM were detected in nematodes, indicating the presence of methyltransferases participating in xenobiotic metabolism. These enzymes may help nematodes deactivate potentially harmful substances^29^.

Glucuronidation (**M9**) of OFM was detected only in ovine liver models, whereas glycosylation (**M10–11**) of OFM was observed only in nematodes (Figs. 3, S4). These results are consistent with previous studies indicating that nematodes primarily biotransform xenobiotics through glycosylation pathways^36,37^. Furthermore, differences in enzyme and substrate availability may explain the divergence in conjugation pathways. Vertebrate livers contain a high number of UDP-glucuronosyltransferase types and necessary cofactors, such as UDP-glucuronic acid, which makes glucuronidation efficient^38^. Conversely, nematodes may possess a broader set of glycosyltransferases tailored for conjugation with endogenous sugars^37^. Furthermore, the presence of OFM conjugates in both nematode homogenate and medium provides additional evidence that nematodes possess functional efflux systems. Therefore, these organisms are capable of not only metabolizing OFM through phase I and phase II reactions, but also actively transporting the resulting conjugates out of the cells. Overall, only small differences were found between strains and sexes. The only exception was **M10**, which was notable in females of the ISE strain (Fig. S4).

## Conclusions

This study highlights the importance of integrating parasite and host metabolism in evaluating new anthelmintic candidates. The first comprehensive characterization of OFM biotransformation in *H. contortus* and in vitro ovine liver models revealed extensive metabolism via phase I and phase II pathways. Our results showed that OFM undergoes diverse biotransformation reactions, including hydroxylation, epoxidation coupled with hydration, loss of a CF_3_ group, glucuronidation, glycosylation, and methylation. Notably, nematodes and ovine liver models share similar phase I oxidative metabolic pathways, likely mediated by CYP enzymes. However, both models exhibit a distinct profile of hydroxylated metabolites. Phase II metabolism differs between hosts and parasites; nematodes primarily employ glycosylation for conjugation, while ovine liver models favored glucuronidation, reflecting species-specific detoxification mechanisms. The capacity of *H. contortus* to metabolize OFM suggests a potential defense mechanism against the drug. Understanding OFM biotransformation pathways is essential for optimizing its use as an anthelmintic. Further in vivo studies, including comprehensive pharmacokinetic characterization and evaluation of the efficacy and safety profiles of OFM in host, are required to confirm these findings.

## Supplementary Information

Below is the link to the electronic supplementary material.


Supplementary Material 1


## Data Availability

All data supporting the results reported in this manuscript are available within the article and its supplementary materials. The data underlying this study are openly available in Zenodo at: https://doi.org/10.5281/zenodo.19651641.
