# Supplementary material for: Biotransformation of obefazimod, a novel potential anthelmintic, in sheep and the target nematode Haemonchus contortus
Source: Sci Rep. 2026 Apr 22;16:18620. doi: 10.1038/s41598-026-49484-1 (PMC13270096; doi:10.1038/s41598-026-49484-1)
Supplement: Supplementary file 1 — Supplementary Material 1 [file 41598_2026_49484_MOESM1_ESM.docx]

**Biotransformation of obefazimod, a novel potential anthelmintic, in sheep and the target nematode *Haemonchus contortus***

**Supplementary information**

**Scientific Reports**

*Lochman Lukáš^a^*^#^*, Novák Martin^b^*^#^*, Skálová Lenka^c^, Svobodová Gabriela^c^, Kučera Radim^a*^ and Raisová Stuchlíková Lucie^c*^*

^a^ Department of Pharmaceutical Chemistry and Pharmaceutical Analysis, Faculty of Pharmacy in Hradec Králové, Charles University, Akademika Heyrovského 1203, 500 05 Hradec Králové, Czech Republic

^b^ Biomedical Research Centre, University Hospital Hradec Králové, Sokolská 581, 50005 Hradec Králové, Czech Republic

^c^ Department of Biochemical Sciences, Faculty of Pharmacy in Hradec Králové, Charles University, Akademika Heyrovského 1203, 500 05 Hradec Králové, Czech Republic

^#^ These authors contributed equally to this work.

**Figure S1.** The fragmentation spectra of the **phase I metabolites M1-6** of obefazimod (OFM; ABX464). The parent OFM metabolite structure is denoted in blue, and the proposed structures of characteristic product ions are indicated in black. Specific product ions that suggest the hydroxylation position in metabolites **M2–M6** are underlined.

**Figure S2.** The fragmentation spectra of the **phase I metabolites M7-8** of obefazimod (OFM; ABX464). The parent OFM metabolite structure is denoted in blue, and the proposed structures of characteristic product ions are indicated in black.

**Figure S3.** The fragmentation spectra of the **phase II metabolites M9-11** of OFM. The parent OFM metabolite structure is denoted in blue, and the proposed structures of characteristic product ions are indicated in black.

**Figure S4.** Relative representation of metabolites formed after incubation with ovine liver models, i.e., ovine hepatocytes and precise-cut liver slices (PCLS), and *H. contortus* nematodes, i.e., Inbred-Susceptible-Edinburg, MHco3 strain (ISE) and multi-resistant White River, MHco4 strain (WR). Metabolite concentrations are expressed as percentages (%), based on the ratio between the metabolite’s peak area and the peak area of the internal standard (IS) monepantel. Values were normalized to 1 mg of protein content. Data are presented as mean ± S.D. (n = 3). The abbreviation ND stands for not detected.

**Table S1.** Analytical description of OFM and its identified metabolites containing details regarding their designation of present metabolites, retention times, theoretical and experimental *m/z* values of [M+H]^+^ in ESI positive-ion mode, mass accuracies, elemental composition, and description of metabolic reaction. Specific product ions that suggest the hydroxylation position in metabolites **M2–M6** are underlined.

| Designation | t_R_ [min] | *m/z* values [M+H]^+^ | | Exact mass shift (ppm) | Elemental composition | Metabolic reaction | | Main fragment ions | | |
| --- | --- | --- | --- | --- | --- | --- | --- | --- | --- | --- |
|  |  | Detected | Calculated |  |  | Phase I | Phase II | Detected *m/z* values | Error (mDa) | Elemental composition |
| OFM | 12.24 | 339.0507 | 339.0507 | 0.00 | C_16_H_11_ClF_3_N_2_O | - | - | 303.0738  270.0550  253.0526  218.0837  180.0209  162.0104  127.0416 | -0.66  -1.48  -0.79  -0.46  -1.11  -0.62  -0.79 | C_16_H_10_F_3_N_2_O  C_15_H_11_ClN_2_O  C_15_H_10_ClN_2_  C_15_H_10_N_2_  C_9_H_7_ClNO  C_9_H_5_ClN  C_9_H_5_N |
| M1 | 7.08 | 271.0632 | 271.0633 | -0.37 | C_15_H_12_ClN_2_O | Loss of CF_3_  ∆ *m/z* = -67.9874 | - | 253.0524  235.0863  218.0839  180.0209  162.0103  127.0415  109.0521 | -1.58  -1.28  0.46  -1.11  -1.23  -1.57  -0.92 | C_15_H_10_ClN_2_  C_15_H_11_N_2_O  C_15_H_10_N_2_  C_9_H_7_ClNO  C_9_H_5_ClN  C_9_H_5_N  C_6_H_7_NO |
| M2 | 10.68 | 355.0455 | 355.0456 | -0.28 | C_16_H_11_ClF_3_N_2_O_2_ | Hydroxylation  Quinoline  ∆ *m/z* = 15.9949 | - | 319.0688  286.0499  269.0474  234.0784  196.0159  178.0054  150.0106  142.0287 | -0.31  -1.75  -1.12  -1.28  -0.51  0.00  0.67  0.00 | C_16_H_10_F_3_N_2_O_2_  C_15_H_11_ClN_2_O_2_  C_15_H_10_ClN_2_O  C_15_H_10_N_2_O  C_9_H_7_ClNO_2_  C_9_H_5_ClNO  C_8_H_5_ClN  C_9_H_4_NO |
| M3 | 10.90 | 355.0455 | 355.0456 | -0.28 | C_16_H_11_ClF_3_N_2_O_2_ | Hydroxylation  Quinoline  ∆ *m/z* = 15.9949 | - | 319.0687  286.0500  269.0474  234.0785  196.0159  178.0053  168.0210  150.0105  143.0366 | -0.63  -1.40  -1.12  -0.85  -0.51  -0.56  -0.60  0.00  0.00 | C_16_H_10_F_3_N_2_O_2_  C_15_H_11_ClN_2_O_2_  C_15_H_10_ClN_2_O  C_15_H_10_N_2_O  C_9_H_7_ClNO_2_  C_9_H_5_ClNO  C_8_H_7_ClNO  C_8_H_5_ClN  C_9_H_5_NO |
| M4 | 11.27 | 355.0455 | 355.0456 | -0.28 | C_16_H_11_ClF_3_N_2_O_2_ | Hydroxylation  Phenoxy ring  ∆ *m/z* = 15.9949 | - | 337.0344  319.0695  269.0472  234.0785  178.0291  162.0104  151.0182 | -1.78  1.88  -1.86  -0.85  -0.56  -0.62  -0.66 | C_16_H_9_ClF_3_N_2_O  C_16_H_10_F_3_N_2_O_2_  C_15_H_10_ClN_2_O  C_15_H_10_N_2_O  C_9_H_7_ClN_2_  C_9_H_5_ClN  C_8_H_6_ClN |
| M5 | 11.62 | 355.0455 | 355.0456 | -0.28 | C_16_H_11_ClF_3_N_2_O_2_ | Hydroxylation  Quinoline  ∆ *m/z* = 15.9949 | - | 319.0688  286.0500  269.0474  234.0783  196.0158  178.0053  168.0208  150.0105  142.0287 | -0.31  -1.40  -1.12  -1.71  -1.02  -0.56  -1.79  0.00  0.00 | C_16_H_10_F_3_N_2_O_2_  C_15_H_11_ClN_2_O_2_  C_15_H_10_ClN_2_O  C_15_H_10_N_2_O  C_9_H_7_ClNO_2_  C_9_H_5_ClNO  C_8_H_7_ClNO  C_8_H_5_ClN  C_9_H_4_NO |
| M6 | 11.73 | 355.0454 | 355.0456 | -0.56 | C_16_H_11_ClF_3_N_2_O_2_ | Hydroxylation  Quinoline  ∆ *m/z* = 15.9949 | - | 319.0688  286.0502  269.0474  233.0707  194.0240  168.0209  150.0104 | -0.31  -0.70  -1.12  -1.29  -0.52  -1.19  -0.67 | C_16_H_10_F_3_N_2_O_2_  C_15_H_11_ClN_2_O_2_  C_15_H_10_ClN_2_O  C_15_H_9_N_2_O  C_9_H_7_ClN_2_O  C_8_H_7_ClNO  C_8_H_5_ClN |
| M7 | 9.00 | 373.0568 | 373.0561 | 1.88 | C_16_H_13_ClF_3_N_2_O_3_ | Epoxidation, hydration  ∆ *m/z* = 34.0054 | - | 355.0456  327.0505  320.0761  292.0819  168.0210 | 0.28  -0.61  -1.87  0.34  -0.60 | C_16_H_11_ClF_3_N_2_O_2_  C_15_H_11_ClF_3_N_2_O  C_16_H_11_F_3_N_2_O_2_  C_15_H_11_F_3_N_2_O  C_8_H_7_ClNO |
| M8 | 9.09 | 373.0561 | 373.0561 | 0.00 | C_16_H_13_ClF_3_N_2_O_3_ | Epoxidation, hydration  ∆ *m/z* = 34.0054 | - | 355.0456  337.0793  327.0506  319.0689  309.0844  291.0738  234.0786  206.0837 | 0.28  -0.30  -0.31  0.31  -0.32  -0.69  -0.43  -0.49 | C_16_H_11_ClF_3_N_2_O_2_  C_16_H_12_F_3_N_2_O_3_  C_15_H_11_ClF_3_N_2_O  C_16_H_10_F_3_N_2_O_2_  C_15_H_12_F_3_N_2_O_2_  C_15_H_10_F_3_N_2_O  C_15_H_10_N_2_O  C_14_H_10_N_2_ |
| M9 | 8.08 | 515.0826 | 515.0827 | -0.19 | C_22_H_19_ClF_3_N_2_O_7_ | - | Glucuronidation  ∆ *m/z* = 176.0320 | 339.0534 | 7.96 | C_16_H_11_ClF_3_N_2_O |
| M10 | 8.45 | 517.0984 | 517.0984 | 0.00 | C_22_H_21_ClF_3_N_2_O_7_ | Hydroxylation  ∆ *m/z* = 15.9949 | Glycosylation  ∆ *m/z* = 162.0528 | 355.0456 | 0.00 | C_16_H_11_ClF_3_N_2_O_2_ |
| M11 | 9.15 | 517.0985 | 517.0984 | 0.19 | C_22_H_21_ClF_3_N_2_O_7_ | Hydroxylation  ∆ *m/z* = 15.9949 | Glycosylation  ∆ *m/z* = 162.0528 | 355.0454 | -0.56 | C_16_H_11_ClF_3_N_2_O_2_ |
| M12* | 12.37 | 369.0611 | 369.0612 | -0.27 | C_17_H_13_ClF_3_N_2_O_2_ | Hydroxylation  ∆ *m/z* = 15.9949 | Methylation  ∆ *m/z* = 14.0156 | - | - | - |
| M13* | 12.54 | 353.0663 | 353.0663 | 0.00 | C_17_H_13_ClF_3_N_2_O | - | Methylation  ∆ *m/z* = 14.0156 | - | - | - |

* M12 and M13 were proposed only based on the exact mass. Confirmation by fragmentation spectra was not possible due to the low concentrations.
